# Clinical signs, MRI findings and outcome in dogs with peripheral vestibular disease: a retrospective study

**DOI:** 10.1186/s12917-020-02366-8

**Published:** 2020-05-25

**Authors:** Rocio Orlandi, Rodrigo Gutierrez-Quintana, Beatrice Carletti, Camilla Cooper, Josep Brocal, Sara Silva, Rita Gonçalves

**Affiliations:** 1 Department of Small Animal Clinical Science, Institute of Veterinary Science, University of Liverpool, Neston, CH64 7TE UK; 2 School of Veterinary Medicine, College of Medical Veterinary and Life Sciences, University of Glasgow, Garscube Estate, 464 Bearsden Road, Glasgow, G61 1QH UK; 3 Wear Referrals Veterinary Hospital, Bradbury, Stockton-on-Tees, TS21 2ES UK

**Keywords:** Peripheral vestibular disease, Idiopathic vestibular disease, Otitis media/interna, MRI, Outcome

## Abstract

**Background:**

Vestibular dysfunction is relatively common in dogs, with a prevalence of 0.08% reported in primary veterinary care in the UK. There are several studies investigating how to differentiate between peripheral and central vestibular disease but only limited information regarding the possible underlying causes for peripheral vestibular dysfunction in dogs. This study therefore aimed to describe the clinical signs, magnetic resonance imaging findings (MRI), underlying causes and outcome in a large population of dogs diagnosed with peripheral vestibular disease.

**Results:**

One hundred eighty-eight patients were included in the study with a median age of 6.9 years (range 3 months to 14.6 years). Neurological abnormalities included head tilt (*n* = 185), ataxia (*n* = 123), facial paralysis (*n* = 103), nystagmus (*n* = 97), positional strabismus (*n* = 93) and Horner syndrome (*n* = 7). The most prevalent diagnosis was idiopathic vestibular disease (*n* = 128), followed by otitis media and/or interna (*n* = 49), hypothyroidism (*n* = 7), suspected congenital vestibular disease (*n* = 2), neoplasia (*n* = 1) and cholesteatoma (n = 1). Long-term follow-up revealed persistence of head tilt (*n* = 50), facial paresis (*n* = 41) and ataxia (*n* = 6) in some cases. Recurrence of clinical signs was observed in 26 dogs. Increasing age was associated with a mild increased chance of diagnosis of idiopathic vestibular syndrome rather than otitis media and/or interna (*P* = 0.022, OR = 0.866; CI 0.765–0.980). History of previous vestibular episodes (*P* = 0.017, OR = 3.533; CI 1.251–9.981) was associated with an increased likelihood of resolution of the clinical signs whilst contrast enhancement of cranial nerves VII and/or VIII on MRI (*P* = 0.018, OR = 0.432; CI 0.251–0.868) was associated with a decreased chance of resolution of the clinical signs.

**Conclusions:**

Idiopathic vestibular disease is the most common cause of peripheral vestibular dysfunction in dogs and it is associated with advanced age. Incomplete recovery from peripheral vestibular disease is common, especially in dogs presenting with cranial nerve enhancement on MRI but less so if there is previous history of vestibular episodes.

## Background

The vestibular system (VS) is the component of the nervous system responsible for maintaining equilibrium and balance [1, 2]. Anatomically, the VS can be divided into peripheral and central components. The peripheral vestibular system is composed of the cranial nerve (CN) VIII (vestibulocochlear nerve) and its receptors which are located in the inner ear. Information collected by the peripheral vestibular system is then transmitted into the central vestibular system which is located inside the brain [3].

There are numerous causes of vestibular dysfunction and problems affecting balance are relatively common in dogs [2], with an overall 0.08% prevalence reported in primary veterinary care in the UK [4]. Vestibular disease was the underlying reason for approximately one third of dogs undergoing advanced imaging of the brain for investigation of a neurological problem at a referral institution [5]. There are now several studies looking at how to differentiate from peripheral and central disease and looking at their possible underlying causes [5–8].

Peripheral vestibular disease (PVD) is a neurological disorder secondary to a lesion affecting the vestibular receptors, ganglion or nerve. The main causes for this are ear disease and idiopathic vestibular syndrome (IVS). The latter is a benign condition that generally resolves spontaneously within a few weeks and for which the underlying cause is yet unknown [2, 8, 9]. Other causes of PVD include toxicity (caused by systemic aminoglycosides or topical chlorhexidine), hypothyroidism (which typically causes polyneuropathy, affecting also the facial nerve) [10–12], congenital vestibular disease and head trauma or neoplasia affecting the petrous portion of the temporal bone or the middle ear [2].

Clinical signs related to vestibular dysfunction are usually unilateral and commonly include loss of balance, asymmetric rolling, leaning or falling ataxia, head tilt, spontaneous positional nystagmus and/or strabismus [3]. Underlying causes, investigation and outcome information remained limited in previous studies of PVD in dogs. The study by Schunk et al. [9] is, to our knowledge, the largest describing clinical data and follow-up evaluations of PVD in the dog. This study, somewhat limited by the use of radiography as their preferred imaging diagnostic technique, found otitis media/interna as the most common cause for PVD. Recently, a study investigating vestibular disease through advanced imaging found IVS as the most common cause of PVD in dogs but this was in a small population of only 25 patients [8].

The main aim of our study was therefore to determine the frequency of the different underlying causes of PVD in dogs and to gather information on long-term outcome of such patients. We hypothesised that there could be specific risk factors associated with the underlying causes and also possibly with extent of recovery and recurrence of clinical signs.

## Results

A total of 188 patients met the inclusion criteria, 73 of them were females (38.8%) and 115 (61.2%) were males with an overall median age of 6.9 years (range 3 months to 14.6 years). The most commonly affected breeds were the Cavalier King Charles spaniel (*n* = 38, 20%), Boxer (*n* = 22, 12%), Cocker spaniel (*n* = 21, 11%), Labrador (*n* = 11, 6%), French bulldog (*n* = 9, 5%) and English springer spaniel (*n* = 8, 4%). One hundred and sixty dogs (85.1%) presented with an acute onset of clinical signs. Previous episodes of vestibular dysfunction were reported in 24 dogs (12.7%), all of which presented with acute-onset symptoms. Clinical signs were non-progressive in 138 (73.4%) patients and 123 of these had an acute onset of vestibular signs. Presenting clinical signs included head tilt in 185 dogs (98.4%), ataxia in 123 dogs (65.4%), facial paralysis in 103 dogs (54.7%), nystagmus in 97 dogs (51.6%) and positional strabismus in 93 dogs (49.5%). Of the patients presenting with facial paralysis, 23 (12.2%) were bilaterally affected. Horner syndrome was identified in 7 patients (3.7%).

**Fig. 1:**
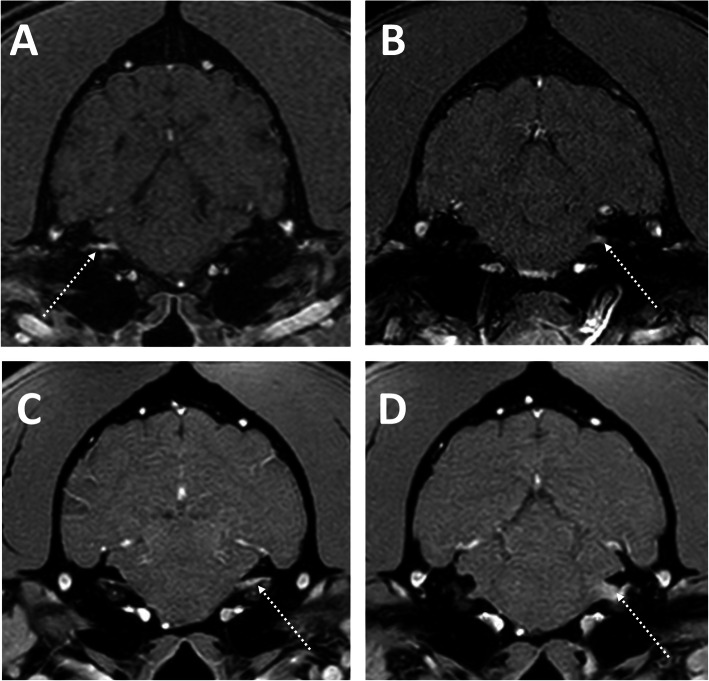
Transverse post-contrast T1-weighted thin-sliced MR images of the caudal brainstem of a dog with **a** right-sided facial nerve enhancement (arrow); **b** left-sided vestibulocochlear nerve enhancement (arrow) and **c** and **d** left-sided simultaneous facial and vestibulocochlear nerves enhancement (arrows)

**Fig. 2:**
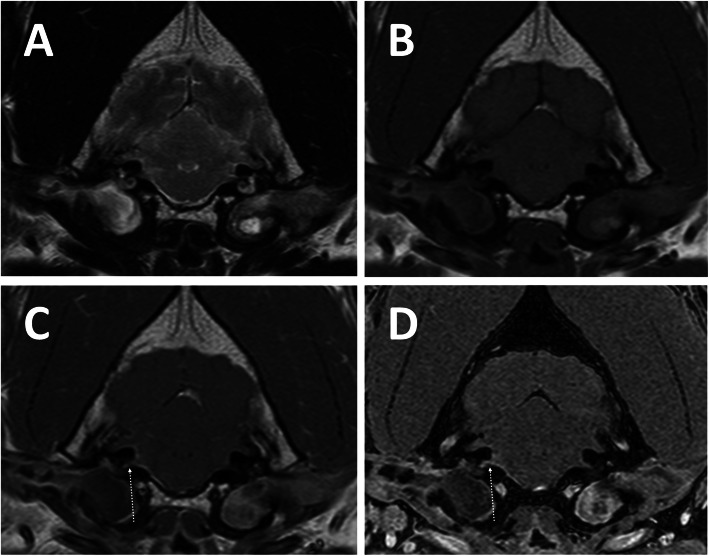
Transverse T2-weighted (**a**), T1-weighted (**b**) and post-contrast T1-weighted (**c**) and volumetric interpolated breath-hold examination (VIBE - **d**) MR images of a dog with bilateral otitis media and externa, right-sided otitis interna and mild meningeal and vestibulocochlear nerve contrast enhancement on the right side (arrows), adjacent to the tympanic bulla

**Fig. 3:**
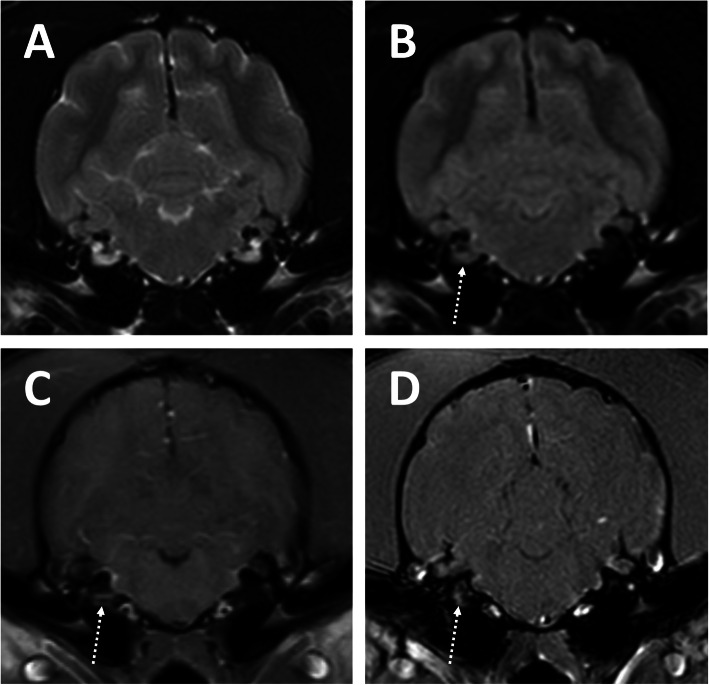
Transverse T2-weighted (**a**), Fluid-Attenuated Inversion Recovery (FLAIR - **b**) T1-weighted post-contrast (**c**) and T1 High Resolution Isotropic Volume Excitation (THRIVE – **d**) MR images of a dog with otitis interna in the absence of obvious otitis media. There is no suppression of the fluid in the right inner ear (**b**), which is contrast enhancing (**c** and **d**) (arrows)

Magnetic resonance imaging was performed in all patients and revealed no abnormalities in 85 (45.2%). The most common abnormalities found were facial and/or vestibulocochlear nerve enhancement after contrast administration (Fig. 1), which were seen in 76 dogs (40.4%). These changes affected the facial nerve only in 49 dogs, the vestibulocochlear nerve only in 10 dogs and both nerves simultaneously in 17 dogs (Table 1). Abnormalities affecting the tympanic bullae and/or inner ear were identified in 49 dogs. These included suspected infectious otitis media and interna in 26 dogs (confirmed with a positive bacterial culture in 22 dogs – Fig. 2), infectious otitis media only in 3 dogs (confirmed with a positive bacterial culture in all dogs) and otitis interna only in 2 dogs (Fig. 3). Eighteen dogs were suspected to have primary secretory otitis media (PSOM) due to the presence of hyperintense T2-weighted material in the tympanic bulla in the absence of contrast enhancement and mucoid material identified on myringotomy (the latter was performed in all but one dog and had supportive cytology and negative bacterial culture). The T2-weighted hyperintense material was present bilaterally in 12 cases and on the affected side only in 6 cases. Finally, MRI revealed an enlargement of the facial nerve ventrolateral to the tympanic bulla (cytology of the lesion was most suggestive of a peripheral nerve sheath tumour) in one case and a mass like lesion in the tympanic bulla and external ear canal suggestive of cholesteatoma in another case (histopathology of the lesion was supportive of the suspected diagnosis).

**Table 1:** Cranial nerve enhancement identified on magnetic resonance imaging

	Facial nerve (*n* = 49)	Vestibulocochlear nerve (*n* = 10)	Facial and vestibulocochlear nerves (*n* = 17)
Idiopathic vestibular syndrome (*n* = 128)	*n* = 30	*n* = 5	*n* = 10
Otitis media/interna (*n* = 49)	*n* = 16	*n* = 4	*n* = 7
Hypothyroidism (*n* = 7)	*n* = 2	*n* = 1	*n* = 0
Neoplasia (*n* = 1)	*n* = 1	*n* = 0	*n* = 0

Thyroid function was assessed in 119 patients (63.3%) and was found consistent with hypothyroidism in 7 dogs (3.7%); 2 of these patients had contrast enhancement of the facial nerve on MRI. Cerebrospinal fluid analysis was performed in 101 dogs (53.7%) and was found to be abnormal in 17 patients. Eleven of these dogs were diagnosed with otitis media and/or interna (2 with suspected PSOM) and 6 were diagnosed with IVS. In 10/11 dogs diagnosed with otitis there was mild to severe mixed pleocytosis (median 26 WBC/μL, range 6 to 2420 WBC/μL), of which 6 had also increased protein (median 0.67 g/l, range 0.34–2.67 g/l). One dog had albuminocytological dissociation (protein 0.8 g/l). Of the dogs with IVS, 2 had mild mixed pleocytosis (7 WBC/μL and 10 WBC/μL) and 4 had albuminocytological dissociation (median protein concentration 0.325 g/l, range 0.28–0.4 g/l). The CSF analysis was repeated in one of the patients with pleocytosis 5 days later and was normal; this patient had a full recovery from the PVD signs over the following days without medical treatment and died of congestive heart failure 3 years later. Repeat CSF analysis was not performed on the other dog with pleocytosis but he recovered quickly over the subsequent days without medical treatment and had no persistent neurological deficits 7 months later.

The most common final diagnosis was idiopathic vestibular syndrome (IVS), which was found in 128 patients (68%). Forty-nine dogs were diagnosed with otitis media and/or interna (26%), hypothyroidism in 7 dogs (4%), congenital vestibular disease in 2 dogs (1%), neoplasia (malignant peripheral nerve sheath tumour of the facial nerve) in one dog (0.5%) and cholesteatoma in 1 dog (0.5%).

Outcome data was available in 148 patients (78.7%) with a median follow-up time of 12 months (range 4 to 96 months). In 77/148 (52%) patients there was persistence of neurological deficits: head tilt in 50 dogs (34.5%); facial paresis in 41 dogs (28.5%) and ataxia in 6 dogs (4.1%). In 26 dogs (17.6%) there was recurrence of the clinical signs at least once over the following 12 months. Clinical and outcome information of dogs diagnosed with IVS are shown in Table 2.

**Table 2:** Clinical and outcome information for dogs diagnosed with idiopathic vestibular syndrome

	Total number of dogs (*n* = 128)
Age	Median 84.5 months (range 15–175)
Sex	Male *n* = 78/128 (61%) Female *n* = 50/128 (39%)
Onset of clinical signs	Acute *n* = 118/128 (92%) Chronic *n* = 10/128 (8%)
Progression of clinical signs	Progressive n = 21/122 (17%) Non-progressive *n* = 94/122 (77%) Waxing/waning *n* = 7/122 (6%)
Neurological abnormalities on examination	Head tilt *n* = 126/128 (98%) Nystagmus *n* = 68/128 (53%) Ataxia *n* = 79/128 (61%) Facial paralysis *n* = 64/128 (50%) Positional strabismus *n* = 61/128 (48%) Horner syndrome *n* = 4/128 (3%)
MRI findings	No abnormalities *n* = 83/128 (65%) CN VII enhancement *n* = 30/128 (23%) CN VIII enhancement *n* = 5/128 (4%) CN VII and VIII enhancement *n* = 10/128 (8%)
CSF abnormalities	Mild pleocytosis *n* = 2/81 (2%) Albuminocytological dissociation = 4/81 (5%)
Outcome information	Complete recovery *n* = 47/94 (50%) Persistence of clinical signs *n* = 47/94 (50%) Facial paralysis *n* = 28/94 (30%) Head tilt *n* = 25/94 (27%) Ataxia *n* = 4/94 (4%)
Recurrence of clinical signs	Recurrence *n* = 19/85 (22%) No recurrence *n* = 66/85 (78%)

On univariable analysis, age, speed of onset of the clinical signs and previous episodes of vestibular dysfunction showed some evidence of association (*P* < 0.25) with the final diagnosis (IVS or otitis media and/or interna). However, on multivariable analysis, age remained as the only significant (*P* = 0.022) factor, with increasing age associated with a mild increased chance of diagnosis of idiopathic vestibular disease rather than otitis media (OR = 0.866; CI 0.765–0.980). A history of previous episodes, progression of clinical signs, facial paralysis and contrast enhancement of cranial nerves VII and/or VIII on MRI showed some evidence of association (*P* < 0.25) with resolution of the clinical signs on univariate analysis. However, on multivariable analysis, only history of previous episodes (*P* = 0.017, OR = 3.533; CI 1.251–9.981) remained associated with an increased chance of resolution of clinical signs whilst contrast enhancement of cranial nerves VII and/or VIII on MRI (*P* = 0.018, OR = 0.432; CI 0.251–0.868) was associated with a decreased chance of resolution of clinical signs.

None of the variables tested were found to be associated with increased chance of recurrence on univariate analysis.

## Discussion

The most common cause of peripheral vestibular disease in dogs in our study, to our knowledge the largest describing this condition, was idiopathic vestibular syndrome (IVS). This differs from the only previous study describing a similar population [9] which found otitis media/interna as the most common cause. This study did not use advanced imaging modalities and it is likely that this may explain the difference in the results.

The cause for IVS in dogs remains undetermined. There are several well-described causes for acute vestibular syndrome in humans and these are generally divided into isolated spontaneous vertigo (most commonly associated with vestibular neuritis or labyrinthitis and cerebellar infarction), recurrent positional vertigo (most commonly associated with benign paroxysmal positional vertigo) or recurrent spontaneous vertigo (most commonly associated with Ménière’s disease and vestibular migraine) [13]. Interestingly, human patients with vestibular neuritis can have contrast-enhancement of the vestibular nerve and inner ear structures [14], similarly to what was seen in some of our dogs. In humans, this condition is thought to be associated with a viral infection or parainfective process [15] and clinical signs resolve spontaneously over a few days. In one study, Canine herpesvirus-1 (CaHV-1) infection of the vestibular labyrinth and vestibular ganglion on post-mortem examinations of dogs without signs of vestibular disease was detected in 17 and 19% dogs respectively but the role of this virus on vestibular disease in the dog remains unclear [16].

More than half of the dogs in the present study had facial nerve dysfunction at the time of presentation. The facial and vestibulocochlear nerves enter the petrous part of the temporal bone through the internal acoustic meatus and are enclosed in a common dural sheath. This common pathway extends into the proximal part of the facial nerve canal, where the nerves lie in close contact [3]. This proximity explains why facial paresis or paralysis (FP) can be concomitant with a lesion affecting the vestibulocochlear nerve and vice versa [1]. Historically, a concurrent vestibular and facial nerve dysfunction was thought more commonly suggestive of middle ear disease [9, 17], however, in our study, approximately two thirds of dogs with PVD and FP had no middle ear abnormalities and were diagnosed with the idiopathic form of the disease. In recent studies, concomitant facial and vestibular neuropathy of unknown origin (FVNUO) has been reported in dogs [18, 19]. Historically, idiopathic vestibular syndrome has mostly been considered in patients with signs of peripheral vestibular dysfunction alone but the current findings and the cases diagnosed with FVNUO [18, 19], support the fact that many patients with no findings on diagnostic investigation and therefore diagnosed under the umbrella term idiopathic syndrome, have concurrent facial nerve dysfunction. In a study with 16 dogs diagnosed with FVNUO, 69% showed albuminocytological dissociation in CSF, suggesting an underlying inflammatory process [19]. Surprisingly, cerebrospinal analysis in the present study, performed in 79 dogs with suspected IVS, revealed only mild mixed pleocytosis in 2 dogs and albuminocytological dissociation in 4 dogs. In human patients presenting with acute facial neuropathy, 15% report associated dizziness [20] and spontaneous nystagmus is also seen in approximately one third of patients with Bell’s palsy (a peripheral-type facial nerve paralysis) most commonly associated with reactivation of herpes simplex virus type 1 [21]. The most common cause for vestibular and concurrent facial nerve impairment in people is a viral inflammation caused by Herpes zoster oticus, which generally also causes severe hearing loss and does not typically resolve with corticosteroids or antiviral drugs [22].

Four dogs diagnosed with IVS and 1 dog with hypothyroidism showed clinical signs compatible with Horner syndrome, which is not usually expected in these conditions. Horner syndrome is caused by interruption of the oculosympathetic pathway, which can result from lesions affecting the first (originating in the hypothalamus and travelling in the tectotegmental spinal tract down to T1-T3), second (exiting spinal cord through T1-T3 nerve roots and forming the thoracic sympathetic trunk which courses within the carotid sheath) and third order neurons (originating at the level of the tympanic bullae and entering the orbita to innervate the smooth dilator muscle of the pupil) [3]. The study by Garosi et al. [17] described that 1/7 dogs with IVS also had Horner syndrome. A breed predisposition for idiopathic Horner syndrome in Golden Retrievers has been reported [23], however, none of our patients were from this breed. Pharmacological testing to attempt to localise the site of the lesion within the oculosympathetic pathway was not performed in the dogs in the current study but this may prove useful in future cases. Unfortunately, the cause for Horner syndrome in patients with idiopathic IVS or hypothyroidism remains obscure.

Magnetic resonance imaging findings in our population revealed that facial and vestibulocochlear enhancement was a commonly found abnormality. In some of these patients, the cranial nerve abnormalities were observed concurrently with otitis media and/or interna. It seems reasonable to hypothesise that these changes were the result of local inflammation associated with the inner and middle ear. In 36% of our patients with clinically identifiable facial nerve dysfunction no detectable abnormalities were identified on MRI. In the study by Smith et al. [18], high-field MRI with 3D sequences showed 86 to 97% sensitivity and 87 to 92% specificity in detecting facial nerve contrast enhancement and provided a better anatomical detail than conventional slices. Hyperintensity following contrast administration in humans with Bell’s palsy is well recognised, found in up to 100% of clinically affected patients [24]. The relatively high percentage of dogs with facial nerve impairment and no detectable facial nerve lesions on MRI in our population is possibly due to the lack of a standardised imaging protocol including thin slice sequences, hampering the correct detection of facial nerve abnormalities. Vestibulocochlear nerve lesions were noted in only 27 dogs, with or without concurrent facial nerve abnormalities. In the study by Smith et al. [18], the sensitivity of 3D sequences for detecting CN VIII abnormalities was significantly lower than for facial nerve changes ranging from 19 to 69%. This would suggest that the nerve may be unaffected and the clinical signs reflect damage to other parts of the vestibular apparatus, that inflammation is less severe and therefore not visible or that the nerve is more difficult to visualise. In humans, it has been hypothesised that the reason for the lack of CN VIII enhancement in patients with idiopathic vestibular neuritis is due to less prominent circumneural blood vessels [25]. In a human study, the use of 4-h delayed high field 3D-FLAIR MR images after double-dose of gadolinium in acute vestibular neuritis has been proven useful in detecting contrast enhancement of the vestibular nerve, however, in 31% of their patients no visible enhancement was observed [14]. This raises the question of whether higher resolution imaging is needed in order to correctly visualise CN VIII lesions in patients with vestibular disorders.

Approximately one quarter of dogs with PVD showed changes compatible with otitis media and/or interna on MRI. In most of these dogs, a suspected diagnosis of otitis media, otitis interna or otitis media/interna was achieved based on what has been defined as imaging characteristics of inflammatory ear disease [1, 2, 26]. Three of those dogs failed to reveal visible MRI changes affecting the inner ear and as it has been previously highlighted, otitis media alone should not result in vestibular signs [2, 17]. Therefore, despite the absence of MRI changes, a degree of inner ear involvement was considered likely in those patients. Cerebrospinal fluid pleocytosis was observed in 10/11 dogs diagnosed with OMI, which suggests there was extension of the infection from the inner ear to the CNS. In most cases of intracranial extension, signs consistent with central vestibular disease are present, although some affected animals may only have signs of peripheral vestibular disease [27]. The remaining 18 dogs with middle ear effusion were diagnosed with primary secretory otitis media (PSOM). PSOM is a disease most commonly described in Cavalier King Charles Spaniels on the basis of cytological and cultural results, although it also affects other breeds in which there is a highly viscous, opaque, greyish or yellowish solid plug appeared to fill the entire middle ear [28]. Dysfunction of the auditory tube is implicated in the pathogenesis of otitis media with effusion (OME) in humans, which may be similar to PSOM in CKCS and in humans may occur secondary to craniofacial abnormalities such as cleft palate [28]. In these dogs, vestibular signs similar to those caused by inflammatory ear disease were observed but it is uncertain whether this condition may have been a concurrent rather than the underlying cause for the PVD signs as this is a common incidental finding in some breeds and it was in most cases bilateral.

In our population, thyroid function was tested in approximately two thirds of the patients and found abnormalities consistent with hypothyroidism in 3.7% of all patients. Hypothyroidism has been associated with peripheral nervous system dysfunction in dogs and humans [29]. In one report on hypothyroidism-induced peripheral neuropathy in the dog, facial nerve paralysis and ataxia were the most common clinical signs [30]. There are two major mechanisms suspected for the development of polyneuropathy in dogs with hypothyroidism. One proposes that decreased mitochondrial ATPase activity impedes the axonal transport of nerves, leading to axonal degeneration and nerve dysfunction. The other suggests that accumulation of xanthomata in the endoneurium and perineurium of peripheral nerves may impair normal nerve function [12]. In our cases, only 2 of the dogs diagnosed with hypothyroidism recovered completely after thyroid hormone administration. Therefore, the relationship between hypothyroidism and peripheral vestibular disease still remains speculative and concurrent IVS cannot be excluded as a possible cause for the vestibular signs.

Outcome information was available in 148 patients with PVD, which represents a significantly larger population that what has been previously reported [6, 7, 9, 19]. Dogs with a previous history of vestibular episodes had an increase likelihood of complete resolution of their clinical signs. This may be related to the underlying condition for these signs as many causes of recurrent vertigo in humans are associated with good outcomes. Contrarily, those with contrast enhancement of cranial nerves VII and/or VIII on MRI were associated with a reduced likelihood of resolution of the clinical signs and this likely is associated with the severity of the inflammatory changes incurred by the nerves. The most frequent persisting clinical sign was the head tilt, which did not resolve in approximately one third of our patients, followed by facial paresis. It is important to note that many of these patients were not examined by a neurologist as the outcome information was collected by telephone conversation with the referring veterinary surgeons or their owners. It is therefore possible that milder deficits may have remained unidentified and that changes such as facial contractures could have been misinterpreted as facial paresis.

Recurrence of clinical signs in dogs with IVS has been previously described in dogs with FVNUO [19]. Similarly, long-term follow-up in our population revealed relapse in 26 dogs (17.6%). No variables associated with recurrence could be identified in the current study, but this may be due to the small number of cases where this occurred. In human patients with acute vestibular syndrome, the underlying causes are typically divided into isolated or recurrent conditions. Relapse of vestibular neuritis is rare (1.9%) but benign paroxysmal positional vertigo, Ménière’s disease and vestibular migraine will often recur with an approximate prevalence of 30–50%, 45–79% and up to 81% respectively [31–33]. It seems likely that dogs diagnosed with IVS may in fact have more than one underlying cause for these signs, therefore explaining the different presentations (mostly related to acute or chronic onset, progression or not of clinical signs and concurrent facial nerve deficits or not), MRI findings (enhancement or not of cranial nerves VII and/or VIII), outcome and recurrence of clinical signs. This raises the question whether a review of the nomenclature currently used, is appropriate as different terms such as IVS, peripheral vestibular syndrome and FVNUO probably all relate to the same group of conditions, which are likely not just one entity but several conditions all manifesting as a similar benign peripheral vestibular and/or facial dysfunction syndrome. Further investigations into each subgroup is warranted to understand the different pathologies better and improve the ability to identify diagnose each specific condition allowing a better ability to prognosticate likelihood of resolution of the clinical signs and recurrence.

The main limitations of this study are those associated with a retrospective investigation with different tests performed as part of the diagnostic investigations and the use of a referral population. It may be postulated that dogs with milder neurological deficits are less likely to be referred and therefore the present study would not represent these dogs appropriately. It is also likely that a proportion of dogs presenting with peripheral vestibular signs and concurrent signs suggestive of otitis media/interna did not undergo MRI and therefore biased the results. This population of dogs nonetheless accurately represents those patients presented to a referral hospital for investigation of peripheral vestibular signs and the results of this study can therefore be used to advise such patients appropriately. Some of the patients included in the otitis media and/or interna group likely had PSOM which may have been a coincidental finding and not the underlying cause of the PVD signs. The authors felt, nonetheless, that changes in the middle ear could not be discarded as a possible cause for the signs, but it is possible that this will be a source of bias. Lastly, all imaging investigations relied on MRI and it can be argued that this modality may not be sensitive in the assessment of certain structures such as the inner ear, so the use of Computed Tomography may have been beneficial in some cases [34].

## Conclusion

Our findings suggest that idiopathic vestibular disease is the most common cause of peripheral vestibular dysfunction in dogs and an association between this condition and older age (compared to patients diagnosed with otitis media/interna) was identified. Incomplete recovery was most commonly seen in patients presenting with cranial nerve enhancement on MRI but a history of previous episodes of vestibular dysfunction was associated with an increased chance of resolution of the clinical signs. Similarly to previously reported, approximately half the dogs presented with peripheral vestibular disease had an incomplete recovery with facial nerve dysfunction and head tilt as the most common persistent clinical signs.

## Methods

Medical records of dogs diagnosed with peripheral vestibular disease at the two contributing institutions between January 2013 and June 2019 were retrospectively reviewed. Data collected included breed, age, sex, duration and speed of onset of the clinical signs, findings of the neurological examination, MRI findings and final diagnosis. Ethical approval for this study was granted by the local Ethics Committee - VREC274 and written consent was obtained from all participants.

The outcome of each case was collected by telephone conversation with the owners or referring veterinary surgeons. Telephone conversation was aimed at determining if the patient recovered completely or there were permanent neurological deficits and if there was reoccurrence of the clinical signs.

Inclusion criteria for this study was a final diagnosis of peripheral vestibular disease and that the patients had undergone magnetic resonance imaging (MRI) of the brain, as well as complete available records for review. Peripheral vestibular dysfunction was defined on the basis of observing one or more of the following clinical signs: vestibular ataxia (asymmetric leaning and falling), head tilt, nystagmus and/or positional strabismus. Dogs were still included if they presented concurrent Horner syndrome (i.e miosis, enophthalmos, protrusion of the third eyelid and/or ptosis) or facial nerve dysfunction, as these abnormalities have been reported in association with PVD [6, 17]. Dogs were diagnosed with suspected central vestibular dysfunction and therefore excluded if they presented with the previously detailed clinical signs but also altered mental status, proprioceptive deficits and/or additional cranial nerve deficits (except for cranial nerve VII). Patients that were initially suspected to have PVD but were later found to have MRI lesions in the central vestibular system were excluded from the study.

MRI examinations were performed using either a 1.5 T (Phillips Intera 1.5 T system, Philips Medical Systems), a 1.5 T (Gyroscan ACS-NT, Philips Medical System) or a 1 T (Magnetom Harmony, Siemens) scanner. The following sequences were obtained in all patients: T2-weighted images (T2W), fluid-attenuated inversion recovery (FLAIR), T2 weighted gradient echo (GE) sequences and pre- and post-contrast (intravenous injection of 0.1 mmol/kg of gadopentetate dimeglumine) T1-weighted images (T1W). Thin slice sequences (T1W gradient echo VIBE-MRI sequences - 1 mm slice thickness or T1W mDIXON - 2 mm slice thickness) post-contrast were also obtained in most but not all patients. All MR images were reviewed by a board-certified radiologist.

When thyroid function assays (thyroxin - T4 and thyroid stimulating hormone - TSH) and routine cerebrospinal fluid (CSF) analysis were performed, the results were also recorded.

Dogs were diagnosed with IVS when the MRI was unremarkable or identified facial and/or vestibulocochlear nerve changes in the absence of other abnormalities. The final diagnosis was otitis media if fluid or soft tissue accumulations were identified in the tympanic bulla on MRI and otitis interna if the inner ear signal failed to suppress on FLAIR images and/or showed contrast enhancement. Hypothyroidism was diagnosed when serum total thyroxine (TT4) and canine thyrotropin hormone (cTSH) concentrations were below and above the reference range, respectively (ie, TT4 < 5 nmol/L and cTSH > 0.5 ng/mL).

Statistical analysis was performed using the software SPSS 22.0 (SPSS Inc., Chicago, Illinois, USA). Continuous data were tested for normality using the Shapiro-Wilk test. Most data were not normally distributed so descriptive statistics were calculated as medians and interquartile ranges. Univariate logistic regression was performed to identify variables associated with incomplete recovery, with recurrence and with final diagnosis (only patients diagnosed with idiopathic vestibular syndrome and otitis media were included in this analysis). Factors hypothesised to be associated with these were: age, speed of onset of the clinical signs (acute < 24 h or chronic ≥24 h), progression or not of the clinical signs, history of similar previous episodes, presence or not of facial paralysis and presence or not of cranial nerve VII and/or VIII contrast enhancement on MRI. Any independent variable demonstrating some association on preliminary univariable analysis (a *P* < 0.25) was considered for inclusion in a multivariable model. Multivariable models were constructed with a manual backwards stepwise removal approach; variables with *P* < 0.05 were retained.

## Data Availability

The datasets used and/or analysed during the current study are available from the corresponding author on reasonable request.

## Abbreviations

MRI: Magnetic resonance imaging
P: *P*-value
OR: Odds ratio
VS: Vestibular system
CN: Cranial nerve
PVD: Peripheral vestibular disease
IVS: Idiopathic vestibular disease
PSOM: Primary secretory otitis media
WBC: White blood cells
CSF: Cerebrospinal fluid
CI: Confidence interval
CaHV-1: Canine herpesvirus 1
FP: Facial paralysis
FVNUO: Facial and vestibular neuropathy of unknown origin
FLAIR: Fluid-attenuated inversion recovery
3D: Three dimensions
OME: Otitis media and interna
CKCS: Cavalier king Charles spaniel
ATPase: Adenosin triphosphatase
T2W: T2 weighted
T1W: T1 weighted
GE: Gradient echo
TSH: Thyroid-stimulating hormone
TT4: Total T4
cTSH: Canine thyroid-stimulating hormone
